# 743. Implementation of Beta-Lactam Therapeutic Drug Monitoring Programs in the Critically Ill: A Multicenter Mixed-Methods Study

**DOI:** 10.1093/ofid/ofac492.034

**Published:** 2022-12-15

**Authors:** Sara Ausman, Kasey Boehmer, Pooja Chitre, Kathleen H Pine, Omar M Abu Saleh, Hafiz Abdul-Aziz, Mohammad H Alshaer, Ognjen Gajic, Paul J Jannetto, Kristin Mara, Lindsay Moreland-Head, Christina G Rivera, Jason Roberts, Andrew D Rule, Marc H Scheetz, Kathryn Shepel, Erin F Barreto

**Affiliations:** Mayo Clinic Health System - Eau Claire, Eau Claire, Wisconsin; Mayo Clinic, Rochester, Minnesota; Arizona State University, Tempe, Arizona; Arizona State University, Tempe, Arizona; Division of Public Health, Infectious Diseases, and Occupational Medicine, Department of Medicine, Mayo Clinic, Rochester, Minnesota; The University of Queensland, Brisbane, Queensland, Australia; University of Florida, Gainesville, Florida; Mayo, Rochester, Minnesota; Mayo Clinic, Rochester, Minnesota; Mayo Clinic, Rochester, Minnesota; Mayo Clinic, Rochester, Minnesota; Mayo Clinic, Rochester, Minnesota; The University of Queensland, Brisbane, Queensland, Australia; Mayo Clinic, Rochester, Minnesota; Midwestern University, Downers Grove, Illinois; Mayo Clinic, Rochester, Minnesota; Mayo Clinic, Rochester, Minnesota

## Abstract

**Background:**

Beta-lactam therapeutic drug monitoring (BL TDM; drug level testing) can facilitate improved outcomes in critically ill patients. Yet less than 20% of hospitals have implemented BL TDM. The purpose of this study was to characterize provider perceptions and key considerations for successful implementation of BL TDM.

**Methods:**

This was a sequential mixed methods study from 2020 to 2021 of stakeholders at three academic medical centers with varying degrees of BL TDM implementation - Mayo Clinic (BL TDM not implemented), University of Florida Health Shands (partially implemented), and Royal Brisbane and Women’s Hospital (fully implemented). Stakeholders completed a survey to characterize their knowledge, perceptions, and experience with BL TDM. A diverse group of 30 respondents were then purposively sampled for semi-structured interviews. Results from the two strands were integrated, themes were identified, and findings were situated within implementation science frameworks.

**Results:**

Among the 138 survey respondents (22% response rate), the majority were physicians (38%) and pharmacists (33%). 71% practiced in critical care and 21% in infectious diseases. The majority of respondents felt BL TDM was relevant to their practice and improved medication effectiveness and safety (Figure 1). Two implementation themes were identified: individual internalization and organizational features. Individuals needed to internalize, make sense of, and agree to BL TDM implementation which was positively influenced by repeated exposure to evidence and expertise. The process of internalization seemed more complex with BL TDM than with other antibiotics (i.e., vancomycin). Organizational considerations relevant to BL TDM implementation included adequate physical and informational infrastructure, access to trained personnel, supportive governance/leadership, and robust process and workflow development.

**Conclusion:**

We found broad enthusiasm about the relevance and potential benefits of BL TDM among stakeholders. Prior literature suggested the primary barrier to implementation was assay availability, but we identified many more individual and organizational attributes which impacted the scale and spread of BL TDM.

**Disclosures:**

**Sara Ausman, PharmD**, Gilead: Honoraria **Christina G. Rivera, PharmD**, Gilead: Grant/Research Support|Gilead: Honoraria|Insmed: Honoraria **Jason Roberts, BPharm(Hons), PhD, FSHP, FISAC**, British Society of Chemotherapy: Grant/Research Support|Cipla: Honoraria|Gilead: Advisor/Consultant|MSD: Advisor/Consultant|MSD: Honoraria|Pfizer: Board Member|Pfizer: Honoraria|Qpex: Grant/Research Support|Sandoz: Board Member|Summit: Advisor/Consultant|Wolters Kluwer: Advisor/Consultant **Marc H. Scheetz, PharmD, MSc**, Abbvie: Advisor/Consultant|Allecra: Grant/Research Support|Merck: Advisor/Consultant|Nevakar: Advisor/Consultant|Nevakar: Grant/Research Support|Premier Healthcare Solutions: Honoraria|Spero: Advisor/Consultant|SuperTrans Medical: Advisor/Consultant|SuperTrans Medical: Grant/Research Support|Takeda: Advisor/Consultant|Third Pole Therapeutics: Advisor/Consultant.

**Figure 1:**
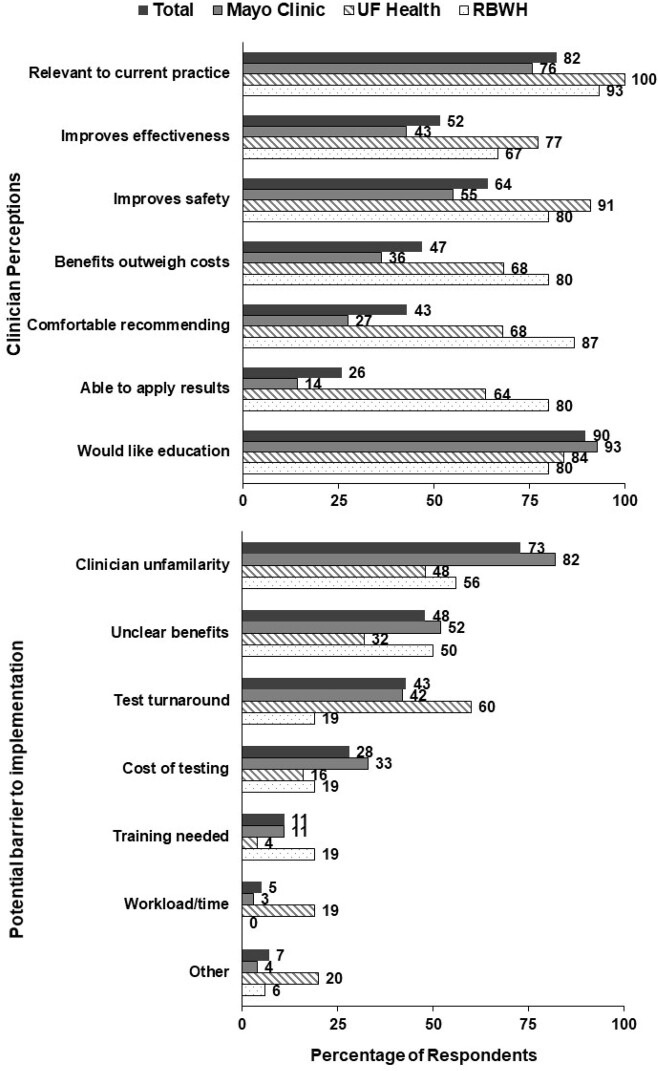
Clinical perceptions about beta-lactam TDM (upper panel) and potential barriers to implementation of beta-lactam TDM (lower panel).

